# Validierung der deutschen Version des Fear-Avoidance Beliefs Questionnaire (FABQ-D) für Patient*innen mit Schulterbeschwerden

**DOI:** 10.1007/s00482-022-00689-z

**Published:** 2023-01-24

**Authors:** Larissa Pagels, Kerstin Lüdtke, Axel Schäfer

**Affiliations:** 1 Institut für Gesundheitswissenschaften, Universität zu Lübeck, Ratzeburger Allee 160, 23562 Lübeck, Deutschland; 2 Fakultät für Soziale Arbeit und Gesundheit, HAWK: Hochschule für angewandte Wissenschaft und Kunst Hildesheim, Hildesheim, Deutschland

**Keywords:** FABQ, Schmerzen, Angst-Vermeidungs-Überzeugungen, Schulterschmerzen, Validität, FABQ, Pain, Fear-avoidance beliefs, Shoulder pain, Validity

## Abstract

**Hintergrund:**

Mit einer Prävalenz von bis zu 30 % sind Schulterbeschwerden das dritthäufigste muskuloskeletale Symptom weltweit. Sowohl die Entstehung als auch der Verlauf wird durch psychosoziale Faktoren, z. B. bewegungsbezogene Angst, beeinflusst. Eines der international gängigsten Messinstrumente zur Erhebung der bewegungsbezogenen Angst ist der Fear-Avoidance Beliefs Questionnaire (FABQ).

**Ziel der Arbeit:**

Untersuchung der Reliabilität (interne Konsistenz) und Validität (Struktur‑, Konstrukt- und prädiktive Validität) des FABQ‑D in einer Population mit Schulterbeschwerden.

**Material und Methoden:**

Im Rahmen einer multizentrischen Querschnittsstudie wurden Proband*innen mit Schulterschmerzen eingeschlossen. Es wurden neben den Angst-Vermeidungs-Überzeugungen die Schmerzintensität, die subjektive Beeinträchtigung im täglichen Leben sowie die Kinesiophobie erfasst. Hierzu dienten der FABQ‑D, die numerische Rating-Skala (NRS), der Shoulder Pain and Disability Index (SPADI) und die Tampa Scale for Kinesiophobia (TSK-GV).

**Ergebnisse:**

Insgesamt konnten 49 Proband*innen (24 Frauen und 25 Männer) mit einem mittleren Alter von 41,8 (SD = 12,8) eingeschlossen werden. Die deskriptive Auswertung auf Itemebene zeigte eine gute interne Konsistenz des FABQ‑D (Cronbachs *α* = 0,88). Die Homogenität der Subskalen variierte dabei stark (Loevingers *H* = 0,66–0,9). Die Korrelationsberechnungen ergaben keine deutliche Konvergenz des FABQ‑D mit der TSK-GV (*r* = 0,3501; *p* = 0,0137). Es konnte eine Divergenz zu den Konstrukten der Messinstrumente NRS (*r* = 0,1818; *p* = 0,2112) und SPADI (*r* = 0,4415; *p* = 0,0015) bestätigt werden. Die Hypothesentestung ergab 42,87 % angenommene Hypothesen und somit keine gute Konstruktvalidität. Es konnte ein signifikanter gemeinsamer Einfluss des FABQ‑D und der TSK-GV auf die Beschwerdedauer festgestellt werden (*R*^*2*^ = 0,3652; *p* ≤ 0,0001). Zudem konnte aufgezeigt werden, dass die größten Einflussfaktoren für einen hohen FABQ-D-Wert die funktionellen Beeinträchtigungen (SPADI) und die Beschwerdedauer bilden (*R*^*2*^ = 0,3066; *p* = 0,0002). Die Subgruppenanalyse zeigte einen signifikant höheren Wert des FABQ‑D bei den älteren Proband*innen (40- bis 65-jährig; *t* = 3,8084/*df* = 47, *p* = 0,0002).

**Diskussion:**

Der FABQ‑D ist ein reliables Messinstrument. Die Konstruktvalidität konnte nur eingeschränkt bestätigt und sollte in zukünftigen Studien weiter untersucht werden. Die Ergebnisse dieser Studie sind vergleichbar mit vorangegangenen Validierungsstudien in anderen Populationen. Der FABQ‑D kann somit als Messinstrument zur Erhebung der bewegungsbezogenen Angst bei Schulterpatient*innen verwendet werden.

## Abstract (en)

**Background:**

With a prevalence of up to 30%, shoulder disorders form the third largest group of musculoskeletal complaints worldwide. Their formation and development are influenced by psychosocial factors such as movement-related fear. One of the internationally most common measurements for quantifying fear of movement is the Fear-Avoidance Beliefs Questionnaire (FABQ).

**Objectives:**

To investigate the reliability (internal consistency) and validity (structural validity, construct validity, predictive validity) of the FABQ‑D in a shoulder pain population.

**Materials and methods:**

Subjects with shoulder pain were included in a multicenter cross-sectional study. In addition to fear-avoidance beliefs, pain intensity, subjective impairment in daily life and kinesiophobia were recorded. To this end, the FABQ‑D, numeric rating scale (NRS), Shoulder and Pain Disability Index (SPADI) and the Tampa Scale for Kinesiophobia (TSK-GV) were used.

**Results:**

A total of 49 subjects (24 women and 25 men) with a mean age of 41.8 years (SD = 12.8) were included. The descriptive evaluation at item level showed good internal consistency of the FABQ‑D (Cronbach’s *α* = 0.88). The homogeneity factor differs significantly between the subscales (Loevinger’s *H* = 0.66–0.9). The correlation analyses did not show any clear convergence of the FABQ‑D with the TSK-GV (*r* = 0.3501; *p* = 0.0137). A divergence to the constructs of the NRS (*r* = 0.1818; *p* = 0.2112) and SPADI (*r* = 0.4415; *p* = 0.0015) were confirmed. The hypothesis testing resulted in 42.87% of the assumed hypotheses and therefore a low construct validity. The FABQ‑D and the TSK-GV showed a significant influence on the duration of the complaints (*R*^*2*^ = 0.3652; *p* ≤ 0.0001). It was also shown that the greatest factors for a high FABQ‑D value were functional impairment (SPADI) and duration of symptoms (*R*^*2*^ = 0.3066; *p* = 0.0002). The subgroup analysis showed a significantly higher FABQ‑D value in older subjects (40–65 years; *t* = 3.8084/*df* = 47, *p* = 0.0002).

**Conclusion:**

The FABQ‑D is a reliable measurement tool. The construct validity should be further investigated in future studies. This study reproduced results from previous studies in other populations. The FABQ‑D appears to be an adequate measurement tool for quantifying fear of movement in patients with shoulder disorders.

## Hintergrund

Die globale Prävalenz von Schulterbeschwerden liegt bei bis zu 30 %, womit sie die drittgrößte Gruppe der muskuloskeletalen Beschwerden ausmachen, nach denen der Halswirbelsäule und Lendenwirbelsäule [4, 10]. Neben dem individuellen Leid der Betroffenen stellen Schulterbeschwerden auch ein erhebliches gesellschaftliches und wirtschaftliches Gesundheitsproblem dar [2, 38].

In der Therapie von chronischen Schulterbeschwerden sollte eine Vielzahl beeinflussender Faktoren berücksichtigt werden [14, 21] – insbesondere psychosoziale Faktoren sind ein signifikanter Chronifizierungsfaktor [19]. Dies können u. a. sozialer Rückzug bei Angststörungen, niedriger sozialer Status, sekundärer Krankheitsgewinn und bewegungsbezogene Ängste sein. Als besonders wichtig haben sich Letztere herausgestellt [7]. Ein bekanntes Modell, welches den Einfluss eines dauerhaft angstgeleiteten Verhaltens auf die Physis und Psyche darstellt, ist das Fear-avoidance-Modell (FAM; [35]). Ein anhaltendes Vermeidungsverhalten bestimmter Bewegungen, bspw. aufgrund von Angst, kann zu körperlicher Dekonditionierung bis hin zur Immobilisierung führen. Auch können weitere psychische und kognitive Folgeerscheinungen aufgrund des Angst-Vermeidungs-Verhaltens entstehen, wie z. B. Katastrophisieren [19].

Auch die Schmerzverarbeitung im nozizeptiven System wird durch Angst und Aufmerksamkeit sensibilisiert, daraus kann eine erhöhte Schmerzempfindlichkeit resultieren. Bereits bestehende kognitive Schemata werden durch Vermeidungsverhalten fixiert und können bei fehlenden gegenteiligen Erfahrungen auch nicht relativiert werden. Der Einfluss der Angst-Vermeidungs-Überzeugungen auf das Bewegungsverhalten wurde bereits vielfach untersucht und nachgewiesen [3, 22, 33].

Ein Erheben von Angst-Vermeidungs-Überzeugungen, beispielsweise durch patientenzentrierte Fragebögen, spielt somit eine wichtige Rolle in der Behandlung von chronischen Schmerzsyndromen. Tagliaferri et al. (2020) stellten in einem Review heraus, dass die Berücksichtigung psychologischer Faktoren, wie der bewegungsbezogenen Angst, bei der Behandlung von chronischen Rückenschmerzen einen deutlichen Mehrwert im Vergleich zur Regelversorgung zeigt [30]. Eines der international gängigsten Messinstrumente zur Erhebung von bewegungsbezogener Angst ist der Fear-Avoidance Beliefs Questionnaire (FABQ; [7, 16]). Neben diesem ist bislang einzig die Tampa Scale for Kinesiophobia (TSK-GV), die ein verwandtes Konstrukt (Kinesiophobie) misst, in die deutsche Sprache übersetzt worden [27]. Beide Skalen beinhalten Items, welche das Vermeidungsverhalten von und Angst vor Bewegungen abfragen. Der FABQ befasst sich allerdings genauer mit den Überzeugungen bezüglich auslösender Ereignisse im Arbeitskontext sowie mit Ängsten bezüglich arbeitsbedingter Bewegungen, weshalb die Untersuchung der Gütekriterien von besonderem Interesse ist [20, 27]. Die Gütekriterien der deutschen Version des FABQ (FABQ-D) sind von Pfingsten et al. bei Patient*innen mit Kreuzschmerzen ermittelt worden [19–21]. Um den FABQ‑D auch bei Patient*innen mit Schulterbeschwerden einsetzen zu können, müssen die Gütekriterien in dieser Population untersucht werden. Das Ziel dieser Studie ist daher die Untersuchung der Gütekriterien des FABQ‑D bei Patient*innen mit Schulterbeschwerden, um eine Empfehlung zum Einsatz des Messinstruments bei dieser Population aussprechen zu können.

## Methode

### Studiendesign und Rekrutierung

Im Rahmen einer multizentrischen Querschnittsstudie fand die Proband*innenrekrutierung von Januar bis Juni 2021 in acht physiotherapeutischen Praxen in Schleswig-Holstein, Hamburg und Niedersachsen statt, welche sich bereit erklärt hatten, an der Studie mitzuwirken.

### Inklusion/Exklusion

Die Inklusionskriterien für die Studie lauteten: Alter < 65 Jahre, Shoulder-Pain-and-Disability-Index(SPADI)-Score von mind. 20 % [9, 12, 26], um eine Beeinträchtigung im Alltag sicherzustellen und ausreichende Deutschkenntnisse (für das Verständnis der Fragen). Folgende Exklusionskriterien wurden formuliert: frisches Trauma (< 5 Tage) im Bereich der Schulter, neurologische Erkrankungen als Ursache für die Schulterbeschwerden. Die Kriterien wurden von den behandelnden Physiotherapeut*innen geprüft.

### Messinstrumente

Der FABQ wurde 1993 von Waddell et al. konstruiert, die deutsche Version wurde 2000 von Pfingsten et al. übersetzt [21, 36]. Der Fragebogen umfasst 16 Items, welche den zum Zusammenhang zwischen Aktivität/Belastung sowie der beruflichen Situation und Rückenschmerzen erfragen. Zum Einsatz bei Schulterpatient*innen wurde der Begriff „Rücken“ analog zur englischen Validierungsstudie mit „Schulter“ ersetzt [6]. Der FABQ‑D beinhaltet drei Subskalen: FABQ‑1 (Beruf und Arbeitstätigkeit sind die Ursache der Schmerzen; Item 6–11), FABQ‑2 (prognostische Annahme der Patient*innen über die wahrscheinliche Wiederaufnahme ihrer Berufstätigkeit; Item 12–16) und FABQ‑3 (Vorstellung der Patient*innen, dass körperliche Aktivität und Schmerzen zusammenhängen; Item 1–5; [12]). Der Fragebogen wird anhand einer 7‑Punkte-Likert-Skala (0 = ich stimme definitiv nicht zu; 6 = ich stimme voll zu) beantwortet. Hierbei bedeuten höhere Werte in den Messinstrumenten jeweils eine größere Ausprägung des abgebildeten Konstrukts. Der Maximalwert des FABQ‑D liegt bei 96.

Erhoben wurden zudem soziodemografischen Daten (s. Tab. 1), die aktuelle Schmerzintensität (Ruheschmerz) anhand einer numerischen 11-Punkte-Rating-Skala (NRS) und die funktionellen Beeinträchtigungen im Alltag mit dem SPADI [2]. Der SPADI besteht aus zwei Subskalen: der Schmerzskala (SPADI-pain), welche das Schmerzgeschehen in unterschiedlichen Situationen erfragt, und der Behinderungsskala (SPADI-disability), welche die Beeinträchtigungen bei einer Auswahl von alltäglichen Handlungen abfragt. Insgesamt besteht er aus 13 Items, welche auf einer Skala von 0 bis 10 beantwortet werden sollen. Der Wert 0 bedeutet dabei „keine Schmerzen“ bzw. „ohne Schwierigkeiten durchführbar“ und der Wert 10 bedeutet „schlimmste Schmerzen“ bzw. „Tätigkeit nicht ausführbar“. Die erreichte Punktzahl wird summiert und in Prozent umgerechnet und beschreibt dann die Schwere der Beschwerden (100 = stärkste Beeinträchtigung; [37]).

**Table 1:** 

Korrelationen des FABQ‑D zu Vergleichsfragebögen	Erwartetes Ergebnis	Ergebnis	Konnte die Hypothese angenommen werden?
FABQ‑D vs. TSK-GV	Sehr starke Korrelation (r > 0,75)	Moderate Korrelation (*r* = 0,3501; *p* = 0,0137)	Nein
FABQ‑D vs. SPADI	Schwache bis moderate Korrelation (*r* ≤ 0,4)	Moderate Korrelation (*r* = 0,4415; *p* = 0,0015)	Ja
FABQ‑D vs. NRS	Schwache bis moderate Korrelation (*r* ≤ 0,4)	Schwache Korrelation (*r* = 0,1818; *p* = 0,2112)	Ja
**Subgruppenanalyse**			
Geschlecht	FABQ-D-Werte der Frauen sind signifikant (*p* ≤ 0,05) höher	Kein signifikanter Unterschied	Nein
Alter	FABQ-D-Werte der 40- bis 65-Jährigen sind signifikant (*p* ≤ 0,05) höher	Werte der Älteren sind signifikant höher	Ja
Beschwerdedauer	Signifikanter Unterschied (*p* ≤ 0,05) der FABQ-D-Werte zwischen chronischen und akuten Proband*innen	Kein signifikanter Unterschied	Nein
Leistungssportler*innen	TSK-GV-Werte der Leistungssportler*innen sind signifikant (*p* ≤ 0,05) höher	Kein signifikanter Unterschied	Nein
**Wie viele Hypothesen konnten angenommen werden?**			**3/7 (42,86** **%)**

*FABQ‑D* deutsche Version des Fear-Avoidance Beliefs Questionnaires, *TSK-GV* deutsche Version der Tampa Scale for Kinesiophobia, *SPADI* Shoulder Pain and Disability Index (deutsche Version), *NRS* numerische Ratingskala

Der FABQ‑D und die Tampa Scale for Kinesiophobia (TSK-GV) wurden erhoben, um die bewegungsbezogene Angst in den Dimensionen Angst-Vermeidungs-Überzeugungen und Kinesiophobie zu erheben [21, 27]. Die TSK-GV ist ein Fragebogen mit 11 Items, welche von den Proband*innen innerhalb von 4 Antwortkategorien beantwortet werden („überhaupt nicht einverstanden“ bis „völlig einverstanden“). Je höher die Punktzahl ist, desto ausgeprägter ist die Kinesiophobie. Man kann zwei Subskalen benennen: die Subskala mit somatischem Fokus (TSK-SF) und die Subskala mit dem Fokus auf der Aktivitätsvermeidung (TSK-AA; [27]). Alle Fragen wurden von den Proband*innen vor ihrer physiotherapeutischen Behandlung selbstständig und schriftlich beantwortet.

### Statistische Verfahren

Die soziodemografischen Daten und der FABQ‑D wurden deskriptiv analysiert. Zudem wurde eine deskriptive Itemanalyse des FABQ durchgeführt. Hierbei wurde die interne Konsistenz ermittelt sowie die konvergente und divergente Validität auf Itemebene analysiert. Die Konstruktvalidität wurde durch die Analyse der Strukturvalidität und durch eine Hypothesentestung bestimmt. Die Hypothesen wurden bezüglich der Korrelationen des FABQ‑D zu den weiteren erhobenen Messinstrumenten (TSK-GV, SPADI, NRS) sowie zu Unterschieden mittels einer Subgruppenanalyse (männlich vs. weiblich, < 40-jährig vs. 40- bis 65-jährig, akut vs. chronisch, Leistungssportler*innen vs. keine Leistungssportler*innen) aufgestellt (Tab. 1). Untersucht wurde die konvergente Validität des FABQ‑D zur TSK-GV, da beide ein ähnliches Konstrukt (Angst-Vermeidungs-Überzeugung/Kinesiophobie) abbilden [7, 16]. Dagegen wurde die divergente Validität zu den Konstrukten körperliche Beeinträchtigung im Alltag (SPADI) und Schmerzintensität (NRS) berechnet, da diese Konstrukte sich deutlich von der Angst-Vermeidungs-Überzeugung unterscheiden [12]. Die Subgruppeneinteilung wurde literaturgeleitet vorgenommen [23]. Hierbei wurden die Schulterbeschwerden bei einer Beschwerdedauer von > 6 Monaten als chronisch angesehen [6] und Proband*innen, welche einen Sport wettkampforientiert durchführten, wurden als Leistungssportler*innen klassifiziert. Hierbei war die Belastung der Schulter während des Sports von Interesse [23]. Bisherige Forschungsergebnisse zeigten, dass das Geschlecht (weiblich), eine erhöhte Beschwerdedauer, ein Alter von 40 bis 65 Jahren und das Ausüben von Leistungssport Faktoren sind, welche Schulterbeschwerden fördern [5, 23, 25]. Zudem wurden Zusammenhänge des FABQ‑D mit weiteren Variablen mittels multipler linearer Regressionsmodelle analysiert. Die statistische Signifikanz der Berechnungen wurde bei *p* ≤ 0,05 festgelegt. Die Auswahl der Berechnungen der Gütekriterien erfolgte nach de Vet et al. (2011; [32]).

Mit dem gleichen Datensatz wurden sowohl die Gütekriterien des FABQ‑D als auch die der TSK-GV analysiert. Die Ergebnisse der Berechnungen zur TSK-GV wurden bereits zuvor publiziert [15]. Im Vorfeld der Studie gab es kein Messinstrument zur Erhebung der bewegungsbezogenen Angst in deutscher Sprache, welches bereits für die Population Schulterbeschwerden validiert wurde, weshalb die Möglichkeit der Berechnungen der konvergenten Konstruktvalidität auf Skalenniveau mittels eines etablierten Messinstruments nicht gegeben war. Dieses Verfahren richtet sich nach den Vorgaben von de Vet et al. [32] und wurde bereits in vorangegangenen Studien so durchgeführt [12].

### Fallzahl

Für die Analyse der konvergenten und divergenten Validität des FABQ‑D wurde die Fallzahl mittels G*Power mit *n* = 47 berechnet. Dem liegen folgende Annahmen zugrunde: Korrelation von r < 0,4 (Power 0,7) für die divergente Validität und r > 0,75 (Power 0,5) für die konvergente Validität. Für das Aufstellen linearer Regressionsmodelle mit drei Kovariablen wurde eine Fallzahl von *n* = 48 berechnet. Dabei wurde eine erklärte Varianz von R^2^ = 0,2 und eine Power von 0,8 angenommen.

## Ethik

Die ethische Unbedenklichkeit wurde von der Kommission für Forschungsethik der Hochschule für angewandte Wissenschaften und Kunst (HAWK) Hildesheim mit Votum vom 21.12.2020 festgestellt. Die Proband*innen wurden schriftlich über die Ziele, Inhalte und Risiken der Studie aufgeklärt und konnten jederzeit ohne Angabe von Gründen abbrechen. Alle Proband*innen haben sich schriftlich zur Teilnahme bereit erklärt.

## Ergebnisse

### Beschreibung der Stichprobe und Messergebnisse

Es konnten entsprechend den Einschlusskriterien 49 Proband*innen rekrutiert werden. Alle Ergebnisse hierzu sind detailliert der Tab. 2 zu entnehmen. Die Schmerzintensität betrug zum Messzeitpunkt im Mittel 3,88 (Min. = 0; Max. = 10; *SD* = 2,02). Die Beeinträchtigungen im Alltag wurden mit einem SPADI-Score von 41,32 % (Min. = 19,23 %; Max. = 76,15 %; *SD* = 15,57 %) bewertet. Der FABQ‑D wurde im Mittel mit *M* = 26,43 (Min. = 4; Max. = 76; *SD* = 18,21) und die TSK-GV mit *M* = 23 (Min. = 12; Max. = 39; *SD* = 6,37) bewertet.

**Table 2:** 

Stichprobe	*n* = 49	*Range*
Alter, *M (SD)*	41,8 (12,8)	17–65
Geschlecht weiblich, *n (%)*	24 (48,98)	–
Operiert, *n (%)*	13 (26,53)	–
Zeit seit Op. in Wochen, *M (SD)*	9,23 (5,6)	2–24
Beschwerdedauer in Monaten, *M (SD)*	32,55 (83,26)	0,5–576
Chronisch, *n (%)*	31 (63,27)	–
Zuvor PT, *n (%)*	16 (32,65)	–
Sport, *n (%)*	42 (85,71)	–
Zeit für Freizeitsport in h/Woche, *M (SD)*	4,86 (3,09)	0–15
Leistungssportler*innen, *n (%)*	8 (16,33)	–
Zeit für Wettkampfsport in h/Woche, *M (SD)*	7,88 (5,49)	0–15
NRS, *M (SD)*	3,88 (2,02)	0–10
SPADI, *M (SD)*	41,32 (15,57)	19,23–76,15
FABQ‑D, *M (SD)*	26,43 (18,21)	4–76
TSK-GV, *M (SD)*	23 (6,37)	12–39

*FABQ‑D* deutsche Version des Fear-Avoidance Beliefs Questionnaires, *TSK-GV* deutsche Version der Tampa Scale for Kinesiophobia, *SPADI* Shoulder Pain and Disability Index (deutsche Version), *NRS* numerische Ratingskala

### Deskriptive Auswertung des FABQ-D auf Itemebene

Sowohl das Item 8 als auch das Item 16 zeigen eine deutliche Neigung der Proband*innen (je 95,92 %), die Antwortkategorie 0 („*stimme definitiv nicht zu*“) zu wählen. Die weitere Verteilung der Antworten auf die einzelnen Antwortkategorien sowie die Ergebnisse zur internen Konsistenz und Homogenität des FABQ‑D zeigt Tab. 3. Das Cronbachs *α* für den FABQ‑D liegt bei *α* = 0,88, für die Subskala FABQ‑1 bei *α* = 0,87, für den FABQ‑2 bei *α* = 0,9 und für den FABQ‑3 bei *α* = 0,66. Im Allgemeinen wird ein Cronbachs *α* von > 0,8 für die interne Konsistenz eines „patient-reported outcome measurement“ (PROM) als gut und von > 0,7 als adäquat angesehen [32]. Die interne Konsistenz der Subskalen FABQ‑1 und -2 ist somit als gut zu bewerten und die des FABQ‑3 als schwach. Die Item-Skala-Konsistenz variiert stark und ist mit H_j_ = 0,1–0,94 sehr schwach bis hoch. Item 13 hat den höchsten Loevinger-Skalierbarkeitskoeffizienten mit *H*_*j*_ = 0,94 und Item 5 den niedrigsten mit *H*_*j*_ = 0,1. Der Loevinger-*H*-Koeffizient beschreibt die Homogenität der Skala und liegt für den FABQ‑1 bei *H* = 0,67, für den FABQ‑2 bei *H* = 0,91 und für den FABQ‑3 bei *H* = 0,28 ([29]; Tab. 3).

**Table 3:** 

	Rate der fehlenden Daten (%)	*n*	Antwortkategorien (%)							α‑Item	Loevinger-Hj-Koeffizient	Anzahl der NS Hjk
			0	1	2	3	4	5	6			
*Item 6*	0,00	49	67,35	2,04	4,08	6,12	10,2	4,08	6,12	0,88	0,46	0
*Item 7*	0,00	49	46,94	12,24	8,16	4,08	12,24	6,12	10,2	0,82	0,73	0
*Item 8*	0,00	49	95,92	–	–	–	–	2,04	2,04	0,86	0,88	0
*Item 9*	0,00	49	79,59	4,08	2,04	8,16	–	2,04	4,08	0,83	0,69	0
*Item 10*	0,00	49	38,78	10,2	10,2	10,2	18,37	4,08	8,16	0,84	0,7	0
*Item 11*	0,00	49	59,18	12,24	2,04	8,16	10,2	2,04	6,12	0,83	0,67	0
*Item* 12	0,00	49	63,27	8,16	4,08	8,16	–	6,12	10,2	0,85	0,93	0
*Item 13*	0,00	49	67,27	8,16	4,08	2,04	–	4,08	14,29	0,85	0,94	0
*Item 14*	0,00	49	71,43	4,08	2,04	4,08	2,04	10,2	6,12	0,85	0,92	0
*Item 15*	2,04	49	81,25	10,42	4,17	2,08	–	2,08	–	0,9	0,79	0
*Item 16*	0,00	49	95,92	–	–	–	–	–	4,08	0,94	0,86	0
*Item 1*	0,00	49	34,69	6,12	4,08	8,16	6,12	8,16	32,65	0,7	0,14	2
*Item 2*	0,00	49	8,16	8,16	8,16	18,37	24,49	14,29	18,37	0,56	0,38	1
*Item 3*	0,00	49	32,65	10,2	2,04	20,41	12,24	8,16	14,29	0,44	0,46	0
*Item 4*	0,00	49	12,24	18,37	6,12	14,29	4,08	12,24	32,65	0,54	0,37	1
*Item 5*	0,00	49	28,57	20,41	–	10,2	16,33	12,24	12,24	0,74	0,1	2

### Konstruktvalidität

#### Strukturvalidität

Für die konvergente Itemvalidität wurde herausgefunden, dass 14/16 Items (87,5 %) einen Korrelationskoeffizienten von *r* > 0,4 mit der Subskala, der sie zugeordnet werden, haben. Es weisen 13/16 Items (81,2 %) einen größeren Korrelationskoeffizienten mit der eigenen Subskala auf als mit den anderen Subskalen. Mit Letzterem wird die divergente Validität auf Itemebene beschrieben. Es wird sowohl die Bedingung der konvergenten als auch der divergenten Validität auf Itemebene erfüllt. Die Bedingungen lauten wie folgt: Ein Korrelationskoeffizient von r > 0,4 des Items zum eigenen Summenscore für die konvergente Validität auf Itemebene wird als gut bewertet. Ebenso ist es bei einer größeren Korrelation mit dem Summenscore der eigenen Subskala als mit dem Summenscore der anderen Subskala für die divergente Validität auf Itemebene ([18]; Tab. 4).

**Table 4:** 

	Fabq_1	Fabq_2	Fabq_3
*Item 6* *Item 7* *Item 8* *Item 9* *Item 10* *Item 11*	**0,482** **0,817** **0,532** **0,687** **0,730** **0,761**	0,360 0,621 0,505 *0,774* 0,577 0,664	0,175 0,247 0,102 0,335 0,187 0,145
*Item 12* *Item 13* *Item 14* *Item 15* *Item 16*	0,757 0,681 0,626 *0,701* 0,421	**0,913** **0,933** **0,917** **0,696** **0,461**	0,353 0,341 0,341 0,328 0,214
*Item 1* *Item 2* *Item 3* *Item 4* *Item 5*	−0,087 0,296 0,298 0,287 0,102	−0,177 0,293 0,437 0,403 *0,317*	***0,186*** **0,540** **0,723** **0,550** ***0,122***

#### Hypothesentestung

Zur Berechnung der konvergenten Validität wurde der FABQ‑D mit der TSK-GV und ihren zwei Subskalen einzeln korreliert. Den höchsten Korrelationskoeffizienten zeigte dabei die Korrelation von FABQ‑3 und TSK-GV mit *r* = 0,4789 (*p* = 0,0005). Dieser Zusammenhang ist als moderat zu bezeichnen.

Die divergente Validität wurde mittels Korrelationsberechnungen des FABQ‑D mit der NRS sowie des FABQ‑D mit dem SPADI berechnet. Hierbei ergab sich ein schwacher, nicht signifikanter Zusammenhang zwischen dem FABQ‑D und der NRS (*r* = 0,1818; *p* = 0,2112). Die Korrelation des FABQ‑D mit dem SPADI-Gesamtwert ist moderat (*r* = 0,4415; *p* = 0,0015; [28]; Tab. 5).

**Table 5:** 

	TSK-GV	TSK-SF	TSK-AA	FABQ‑D	FABQ‑1	FABQ‑2	FABQ‑3	NRS	SPADI	SPADI-Schmerz
*TSK-GV*	**1**	**–**	**–**	**–**	**–**	**–**	**–**	**–**	**–**	**–**
*TSK-SF*	**0,8590** 0,0000	**1**	**–**	**–**	**–**	**–**	**–**	**–**	**–**	**–**
*TSK-AA*	**0,9215** 0,0000	**0,5926** 0,0000	**1**	**–**	**–**	**–**	**–**	**–**	**–**	**–**
*FABQ‑D*	**0,3501** 0,0137	**0,3971** 0,0047	0,2495 0,0838	**1**	**–**	**–**	**–**	**–**	**–**	**–**
*FABQ‑1*	0,2183 0,1319	0,2139 0,1400	0,1811 0,2131	**0,8434** 0,0000	**1**	**–**	**–**	**–**	**–**	**–**
*FABQ‑2*	0,1530 0,2939	0,2620 0,0690	0,0420 0,7746	**0,8778** 0,0000	**0,7242** 0,0000	**1**	**–**	**–**	**–**	**–**
*FABQ‑3*	**0,4789** 0,0005	**0,4877** 0,0004	**0,3834** 0,0065	**0,6722** 0,0000	0,2719 0,0587	**0,369** 0,0091	**1**	**–**	**–**	**–**
*NRS*	0,1216 0,4052	0,0541 0,7122	0,1503 0,3027	0,1818 0,2112	0,0386 0,7920	0,0314 0,8303	**0,3792** 0,0072	**1**	**–**	**–**
*SPADI*	0,2571 0,0745	0,2006 0,1670	0,2524 0,0802	**0,4415** 0,0015	0,1768 0,2242	**0,4333** 0,0019	**0,4602** 0,0009	**0,4491** 0,0012	**1**	**–**
*SPADI-Schmerz*	**0,3201** 0,0250	**0,3157** 0,0271	0,2640 0,0668	**0,2847** 0,0474	0,0566 0,6993	0,2115 0,1445	**0,4294** 0,0021	**0,5623** 0,0000	**0,768** 0,0000	**1**
*SPADI-Behinderung*	0,1310 0,3696	0,0665 0,6498	0,1556 0,2857	**0,4366** 0,0017	0,2059 0,1557	**0,4728** 0,0006	**0,3755** 0,0078	0,2697 0,0609	**0,883** 0,0000	**0,3975** 0,0047

*FABQ‑D* deutsche Version des Fear-Avoidance Beliefs Questionnaires, *TSK-GV* deutsche Version der Tampa Scale for Kinesiophobia, *TSK-SF* Subskala mit somatischem Fokus, *TSK-AA* Subskala zur Activity Avoidance, *SPADI* Shoulder Pain and Disability Index (deutsche Version), *NRS* numerische Ratingskala

In der Subgruppenanalyse fand sich ein signifikanter Unterschied im Vergleich der Altersgruppen der unter 40-Jährigen und der 40- bis 65-Jährigen. Die Älteren zeigten dabei einen höheren FABQ-D-Wert an (*t* = 3,8084/*df* = 47, *p* = 0,0002).

Die Konstruktvalidität kann mit 42,87 % angenommener Hypothesen nicht als gut bewertet werden ([24]; Tab. 1).

### Regression

Für die Berechnung der Einflussfaktoren auf den FABQ-D-Wert wurde ermittelt, dass das Modell mit den Prädiktoren SPADI und Beschwerdedauer die höchste erklärte Varianz mit* R*^*2*^ = 0,3066 (*p* = 0,0002) aufwies.

Es wurden verschiedene Regressionsmodelle aufgestellt, um zu ermitteln, wie verschiedene Prädiktoren die Dauer der Schulterbeschwerden beeinflussen. Das Modell, welches die Variablen FABQ‑D, TSK-GV, SPADI und NRS einschloss, zeigte mit einem *adj. R*^*2*^ von 0,22 den besten Wert. Das Regressionsmodell zeigt eine erklärte Varianz von *R*^*2*^ = 0,2854 bei einer Signifikanz von *p* = 0,0045. Hier konnte herausgefunden werden, dass die Prädiktoren FABQ‑D (*t* = 2,13; *p* = 0,038) und TSK-GV (*t* = 2,5; *p* = 0,016) einen statistisch signifikanten Einfluss auf die Beschwerdedauer haben. Es konnte bei der Analyse der prognostischen Faktoren eine moderate Korrelation zwischen dem FABQ‑D und der TSK-GV festgestellt werden. Entsprechend wurde ein Regressionsmodell aufgestellt, bei welchem die Interaktion des FABQ‑D mit der TSK-GV berücksichtigt wurde. Dieses Regressionsmodell ist mit *p* ≤ 0,0001 signifikant und weist eine erklärte Varianz von *R*^*2*^ = 0,3652 auf. Der Prädiktor FABQ-D+TSK-GV (unter Berücksichtigung der Interaktion der Variablen) ist mit *t* = 5,2 (*p* ≤ 0,0001) ein eindeutig signifikanter Einflussfaktor für die Dauer der Schulterbeschwerden (Tab. 6).

**Table 6:** 

Abhängige Variable	Prädiktoren	*Koeffizienten*	*p‑Wert*	*R*^*2*^	*p*
*FABQ‑D*	Beschwerdedauer SPADI	0,0737 0,4667	0,009 0,002	0,3066	0,0002
*Beschwerdedauer*	TSK-GV#FABQ‑D	0,0918	0,000	0,3652	0,0000

*FABQ-D* deutsche Version des Fear-Avoidance-Beliefs Questionnaire, *TSK-GV* deutsche Version der Tampa Scale for Kinesiophobia, *SPADI* Shoulder Pain and Disability Index

## Diskussion

Schmerzgeschehen werden durch psychische Wirkfaktoren begünstigt. Vor allem bei der Chronifizierung von Schmerzen stehen diese inzwischen im Fokus der Forschung. Das Fear-avoidance-Modell bietet einen Erklärungsansatz für die sich bedingenden Faktoren zur Chronifizierung [34]. Die Betroffenen vermeiden Bewegungen, welche die Schulter beanspruchen, aus Angst vor Schmerzen [4, 31]. Das Erheben von Angst-Vermeidungs-Überzeugungen durch den FABQ‑D kann dazu beitragen, dass diese Wirkfaktoren für die Beschwerden der Patient*innen von den Behandelnden erfasst werden und somit in die Behandlung integriert werden können.

Die interne Konsistenz des FABQ‑D kann mit *α* = 0,88 als gut bewertet werden. Dennoch lässt die hohe interne Konsistenz vermuten, dass ein Ausschluss von Items aus der Skala vorgenommen werden könnte, um diese effizienter zu machen [32]. Hier würden sich die Items 5, 8 und 16 aus den in den Ergebnissen genannten Gründen anbieten. Eine Umstrukturierung der Subskalen oder ein Ausschluss dieser Items wären mögliche Schritte.

Die A‑priori-Hypothesen konnten nicht ausreichend bestätigt werden. Dies ist ein Hinweis auf eine geringe Konstruktvalidität des FABQ‑D. Erklärt werden kann dies durch die ambitionierte Formulierung der Hypothesen in Bezug auf die Höhe des Korrelationskoeffizienten. Die Ergebnisse der Subgruppenanalyse überraschen, da sie konträr zur bestehenden Literatur sind [5, 23, 25]. Interessant ist der Einfluss der körperlichen Beeinträchtigung (gemessen am SPADI) und der Beschwerdedauer auf die Bewertung des FABQ‑D. Beide Prädiktoren haben einen signifikanten Einfluss auf die Angst-Vermeidungs-Überzeugungen. Hinzu kommt der deutliche Einfluss der bewegungsbezogenen Angst, erhoben in den Dimensionen Angst-Vermeidungs-Überzeugungen (FABQ-D) und Kinesiophobie (TSK-GV), auf die Dauer der Beschwerden der Proband*innen.

Die Ergebnisse dieser Studie sind vergleichbar zu vorangegangen Studien bei denen die Reliabilität (interne Konsistenz) und Validität des FABQ‑D sowie der englischsprachigen Version untersucht wurden [12, 21]. Verglichen mit der Studie von Pfingsten et al. (1997), die den FABQ‑D an einer Population mit Kreuzschmerzen (LBP) untersuchte, war die Stichprobe dieser Studie deutlich kleiner (LBP: *n* = 87; Schulter: *n* = 49; [21]). Die mittlere Schmerzintensität der Proband*innen war zudem bei der Studie von Pfingsten et al. höher (LBP: 6,4; Schulter: 3,88) und die Dauer der Beschwerden erheblich länger (LBP: 146,8 Monate; Schulter: 32,55 Monate). Die Werte der internen Konsistenz sind vergleichbar (LBP: *α* = 0,85; Schulter: *α* = 0,88). In beiden Studien zeigten sich die Divergenz zur Schmerzintensität. Außerdem zeigten sich Zusammenhänge zwischen der körperlichen Beeinträchtigung sowie der Schmerzdauer und der Bewertung des FABQ‑D. Im Gegensatz zur LBP-Studie konnte in dieser Studie ein Alterseffekt dargestellt werden. Es bleibt offen, ob Schulterbeschwerden und Rückenschmerzen in Bezug auf psychologische Einflussfaktoren vergleichbar sind [39].

Der FABQ‑D scheint, auf Grundlage der vorgestellten Gütekriterien, ein geeigneteres Messinstrument zur Erhebung der bewegungsbezogenen Angst bei Schulterbeschwerden zur sein als die TSK-GV [15]. International werden zur Erfassung bewegungsbezogener Ängste vor allem die Fragebögen FABQ und TSK eingesetzt [16]. Vergleicht man die psychometrischen Eigenschaften der deutschen Versionen in Bezug auf Schulterbeschwerdepatient*innen, so zeigt sich eine Überlegenheit des FABQ‑D. Der FABQ‑D (*α* = 0,88) weist eine höhere interne Konsistenz auf als die TSK-GV (*α* = 0,81). Des Weiteren konnte für den FABQ‑D sowohl die divergente als auch die konvergente Validität auf Itemebene bewiesen werden. Die TSK-GV erfüllt die Bedingungen der konvergenten Validität auf Itemebene nicht und weist somit eine geringere Strukturvalidität auf als der FABQ‑D. Beide Fragebögen können im Rahmen der Hypothesentestung keine gute Konstruktvalidität aufweisen, wobei der Wert des FABQ‑D mit 42,87 % angenommener Hypothesen deutlich über dem der TSK-GV (28,57 %) liegt. Interessant ist, dass die Regressionsmodelle gezeigt haben, dass der FABQ‑D sich vor allem von der Beschwerdedauer, aber auch von der funktionellen Beeinträchtigung (SPADI-Wert) beeinflusst wird. Die TSK-GV wird am stärksten durch die Beschwerdedauer beeinflusst [17].

Der FABQ‑D fragt das Konstrukt der Angst-Vermeidungs-Überzeugungen ab, wohingegen das Konstrukt der TSK-GV die Kinesiophobie ist. Inwieweit die Konstrukte vergleichbar sind, bleibt zu diskutieren. Es kann aber behauptet werden, dass beide Konstrukte (übergeordnet) bewegungsbezogene Ängste erfassen [7, 11].

Die Überlegenheit des FABQ gegenüber der TSK zur Erfassung der bewegungsbezogenen Angst bei Schulterbeschwerden stellten bereits Mintken et al., welche die englischsprachigen Versionen bei Schulterbeschwerden verglichen, dar [12].

In der Zukunft wäre eine weitere Untersuchung der psychometrischen Eigenschaften des FABQ‑D sinnvoll. Interessant wäre hier neben den in dieser Studie bereits durchgeführten Berechnungen auch die Analyse der Änderungssensitivität. Hierbei sollten vor allem weitere Populationen (z. B. Nackenschmerzen) in Betracht gezogen werden. Insbesondere eine Untersuchung der Zusammenhänge zu weiteren psychologischen Messinstrumenten wäre hier ein naheliegender nächster Schritt, um das beste Messinstrument für den klinischen Einsatz herauszuarbeiten. Die Ergebnisse der vorliegenden Studie zeigen, dass der FABQ‑D zur Erhebung der Angst-Vermeidungs-Überzeugungen bei Patient*innen mit Schulterbeschwerden geeignet ist, auch wenn die Ergebnisse aufgrund der eingeschränkten Konstruktvalidität vorsichtig zu bewerten sind. Der FABQ‑D ist ein ökonomisches Messinstrument, welches von den Patient*innen selbständig ausgefüllt werden kann und in der Praxis ergänzend zur Untersuchung bei chronischen Schulterbeschwerden eingesetzt werden kann. Er dient dazu, Angst-Vermeidungs-Überzeugungen, welche zur Chronifizierung von Beschwerden beitragen, herauszuarbeiten und darauf aufbauend eine passende, individualisierte Behandlung aufzubauen.

Der Einfluss psychischer Wirkfaktoren auf die Beschwerden der Patient*innen sollte auch in weiterführenden Studien untersucht werden, da diese im Praxisalltag immer häufiger erhoben und stärker berücksichtigt werden [8, 21]. In dieser Studie konnte dargelegt werden, dass das Ausmaß der Angst-Vermeidungs-Überzeugungen signifikant mit der Beschwerdedauer zusammenhängt. Dabei könnte sich der Einsatz von „behaviour change techniques“ (BCT) oder anderen verhaltensorientierten Maßnahmen in der Therapie positiv auf das Behandlungsergebnis der Betroffenen auswirken [1]. Um eine bestmögliche Betreuung der Betroffenen zu gewährleisten, wäre ein verstärkter interdisziplinärer Ansatz (z. B. Physiotherapie und Psychotherapie) möglich. Die Literatur zeigt, dass es einen deutlichen Mehrwert in der Versorgung chronischer Schmerzpatient*innen durch den Einbezug psychologischer Faktoren gibt [30].

## Limitationen

Limitation der Studie ist die für die durchgeführten Berechnungen zu kleine Stichprobe [13]. Die Aussagekraft der Studie würde durch eine größere Stichprobe gestärkt werden. Dennoch konnten mit der Stichprobe signifikante Ergebnisse erzielt werden, aus denen Erkenntnisse für weitere Forschung gewonnen werden können. Eine weitere Limitation ist, dass mit dem gleichen Datensatz auch die Gütekriterien der TSK-GV für Schulterbeschwerden ermittelt wurden. Es sei darauf hingewiesen, dass es bislang kein validiertes Messinstrument gibt, welches die bewegungsbezogene Angst von Patient*innen mit Schulterbeschwerden besser erfasst als der FABQ‑D.

## Schlussfolgerung

Der Einsatz des FABQ‑D ist zur Erhebung der bewegungsbezogenen Angst bei Patient*innen mit Schulterbeschwerden möglich. Der FABQ‑D stellt ein ökonomisches Messinstrument mit akzeptablen Gütekriterien dar. Das Erheben von Angst-Vermeidungs-Überzeugungen sollte insbesondere in der Untersuchung der Betroffenen von den Behandelnden berücksichtigt werden, um passende, spezifische Maßnahmen in der Therapie anwenden zu können bzw. eine interdisziplinäre Therapie frühzeitig anstreben zu können [10]. Es bedarf weiterer Forschung, um die deutsche Version des FABQ auch für weitere Populationen zu validieren.

## Fazit für die Praxis

- Der FABQ‑D kann als PROM im Rahmen der Therapie eingesetzt werden.
- Der FABQ‑D zeigt das Potenzial, die Prognose von chronischen Schulterbeschwerden zu schärfen.
